# Postoperative nausea and vomiting: current concepts, management strategies, and future perspectives

**DOI:** 10.3389/fphar.2026.1930252

**Published:** 2026-09-11

**Authors:** Lina Qiu, Ahmad Alhaskawi, Safwat Adel Abdo Moqbel, Huadi Yuan

**Affiliations:** 1 Department of Nursing, The Second Affiliated Hospital Zhejiang University School of Medicine, Hangzhou, China; 2 Department of Orthopedics, The First Affiliated Hospital of Zhejiang University School of Medicine, Hangzhou, China; 3 Department of Emergency, The Second Affiliated Hospital of Zhejiang University School of Medicine, Hangzhou, China

**Keywords:** antiemetic prophylaxis, enhanced recovery after surgery, perioperative care, postoperative nausea and vomiting, risk stratification

## Abstract

Postoperative nausea and vomiting (PONV) remains one of the most common and distressing adverse events after anesthesia and surgery, with important effects on patient comfort, recovery quality, discharge readiness, and healthcare resource use. Although advances in anesthetic techniques, antiemetic pharmacology, and enhanced recovery pathways have improved perioperative care, PONV continues to occur frequently because of its multifactorial pathophysiology and variable individual susceptibility. This narrative review provides a clinically oriented overview of current concepts in PONV, including epidemiology, clinical burden, neurochemical mechanisms, risk factors, prediction models, baseline risk reduction, pharmacological prophylaxis, rescue treatment, postdischarge care and future perspectives. PONV results from complex interactions among central and peripheral emetic pathways, including gastrointestinal vagal afferents, the chemoreceptor trigger zone, vestibular input, higher cortical and limbic modulation, and multiple neurotransmitter systems such as serotonin, dopamine, histamine, acetylcholine, substance P, and opioid-mediated signaling. Structured risk assessment, particularly using simplified prediction tools such as the Apfel score, remains essential for guiding individualized prophylaxis. Preventive strategies should combine reduction of modifiable perioperative risk factors with appropriate multimodal antiemetic therapy. Key interventions include minimizing exposure to volatile anesthetics and nitrous oxide when suitable, using opioid-sparing analgesia, optimizing hydration and fasting practices, and integrating regional or neuraxial techniques when appropriate. Pharmacological management relies on agents targeting different receptor systems, including 5-HT3 receptor antagonists, dopamine receptor antagonists, neurokinin-1 receptor antagonists, glucocorticoids, anticholinergics, and antihistamines. Future research should improve prediction accuracy, clarify optimal prophylactic combinations, strengthen guideline implementation, evaluate postdischarge symptoms, and explore artificial intelligence-assisted individualized prevention. A comprehensive, risk-based, and patient-centered approach remains central to reducing the burden of PONV.

## Introduction

Postoperative nausea and vomiting (PONV) is a common adverse event after anesthesia and surgery and continues to represent an important challenge in perioperative medicine. Despite advances in anesthetic techniques, antiemetic pharmacology, and enhanced recovery protocols, PONV remains clinically relevant because of its high incidence, multifactorial etiology, and considerable effect on patient comfort and satisfaction (Benhamou, 2023; Gan et al., 2014). For many patients, nausea and vomiting postoperative are among the most unpleasant aspects of the perioperative experience and may be perceived as highly distressing. The importance of PONV extends beyond temporary discomfort. Persistent nausea and vomiting may delay oral intake, interfere with mobilization, prolong recovery room observation, increase healthcare costs, and contribute to unplanned admission after ambulatory surgery (Jin et al., 2020; Bell et al., 2024). In selected patients, severe vomiting may also increase the risk of wound dehiscence, bleeding, aspiration, dehydration, and electrolyte imbalance (Hocking et al., 2013; Ghosh et al., 2020; Eberhart et al., 2002). Therefore, effective prevention and management of PONV are essential components of patient-centered perioperative care. The pathogenesis of PONV involves complex interactions among the central nervous system, gastrointestinal tract, vestibular system, and chemoreceptor trigger zone. Multiple neurotransmitter pathways contribute to the emetic response, including serotonin, dopamine, histamine, acetylcholine, and substance P acting through neurokinin-1 receptors (Zhang and Wang, 2025; Stoops and Kovac, 2020; Horn et al., 2014). This biological complexity explains why single-agent prophylaxis may be insufficient in patients with moderate or high risk and supports the use of multimodal antiemetic strategies.

This review aims to provide an updated and clinically oriented synthesis of PONV, encompassing its epidemiology, pathophysiology, risk stratification, baseline risk reduction, pharmacological prophylaxis, rescue treatment, and postdischarge management. Particular emphasis is placed on linking validated risk assessment with multimodal preventive strategies, appropriate antiemetic selection, and management of breakthrough symptoms after prophylaxis failure. The review also considers current limitations in risk prediction and implementation of guideline-based care, together with emerging approaches such as artificial intelligence-assisted prediction, to support more individualized perioperative management and identify priorities for future research.

## Methodology

This study was conducted as a non-systematic narrative review of the current evidence on postoperative nausea and vomiting (PONV). Relevant literature was identified through searches of PubMed/MEDLINE and Web of Science. Search terms included “postoperative nausea and vomiting,” “PONV,” “postoperative nausea,” “postoperative vomiting,” “pathophysiology,” “risk factors,” “risk prediction,” “Apfel score,” “antiemetic prophylaxis,” “multimodal prophylaxis,” “rescue treatment,” “5-HT3 receptor antagonists,” “dopamine receptor antagonists,” “neurokinin-1 receptor antagonists,” “dexamethasone,” “scopolamine,” “total intravenous anesthesia,” “opioid-sparing analgesia,” “enhanced recovery after surgery,” “postdischarge nausea and vomiting,” “artificial intelligence,” and “machine learning.” These terms were used individually and in relevant combinations. Additional studies were identified from the reference lists of key guidelines, systematic reviews, meta-analyses, and major clinical studies. Studies were selected according to their relevance to the epidemiology, pathophysiology, risk stratification, prevention, and management of PONV. As this was a narrative review, no formal risk-of-bias assessment or quantitative evidence synthesis was performed.

### Epidemiology and clinical burden of postoperative nausea and vomiting

PONV remains one of the most frequent adverse events following anesthesia and surgery. Although its reported incidence varies according to patient characteristics, surgical population, anesthetic technique, prophylactic strategy, and outcome definition, PONV affects approximately 30% of the general surgical population and may occur in up to 80% of patients at high risk (Apfel et al., 2012a; Gan, 2006; Franck et al., 2010). This high frequency makes PONV a persistent concern in routine perioperative practice despite advances in anesthetic care, antiemetic pharmacology, and enhanced recovery protocols. The clinical relevance of PONV is not limited to its incidence. For many patients, nausea and vomiting are among the most distressing postoperative symptoms and may negatively influence their overall perception of surgical care. In ambulatory and short-stay surgery, where early mobilization, oral intake, and timely discharge are central objectives, even mild or moderate PONV can interfere with recovery milestones (Bell et al., 2024; Chung and Mezei, 1999). As a result, PONV is increasingly recognized as an important patient-centered outcome rather than a minor postoperative inconvenience. PONV can also contribute to measurable postoperative morbidity. Repeated vomiting may cause dehydration, electrolyte imbalance, increased postoperative pain, wound tension, bleeding, hematoma formation, and; in severe cases or vulnerable patients, it may also increase the risk of aspiration or wound dehiscence. Persistent symptoms may delay oral medication intake, nutrition, mobilization, and discharge readiness (Chung and Mezei, 1999; Wesmiller et al., 2017). These consequences are particularly important in patients undergoing procedures where increased intra-abdominal, intrathoracic, or wound pressure may compromise surgical outcomes. From a healthcare-system perspective, PONV increases the demand for postoperative monitoring, nursing care, rescue antiemetic therapy, and prolonged post-anesthesia care unit observation (Parra-Sanchez et al., 2012; Habib et al., 2006). Therefore, PONV has implications not only for patient comfort but also for perioperative efficiency, healthcare utilization, and cost.

### Pathophysiology and neurochemical basis of postoperative nausea and vomiting

**FIGURE 1:**
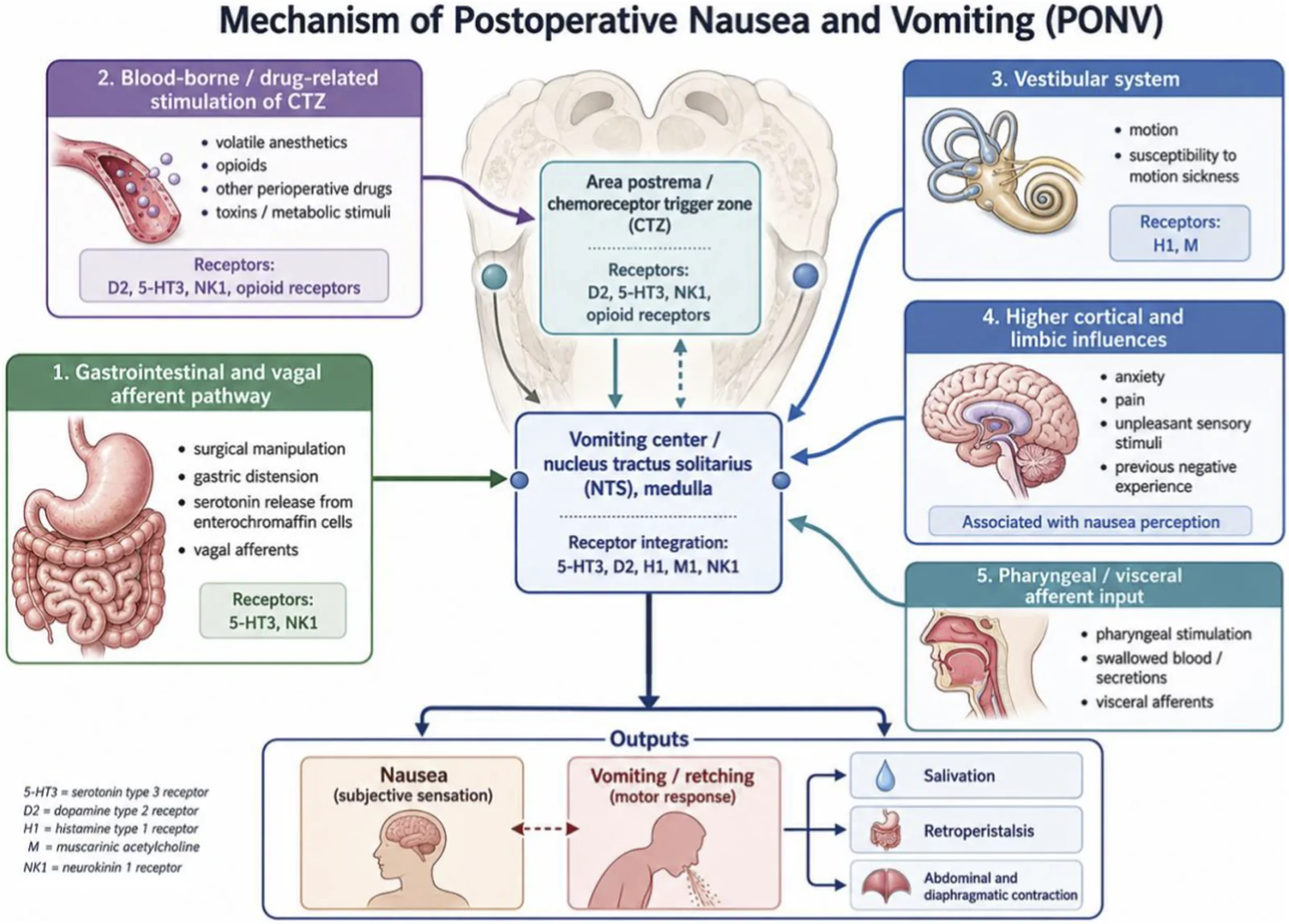
Mechanisms of postoperative nausea and vomiting (PONV). Peripheral and central emetogenic inputs converge on the chemoreceptor trigger zone (CTZ) and nucleus tractus solitarius (NTS), activating key receptor pathways that mediate nausea and vomiting.

The pathophysiology of PONV is complex and involves coordinated interactions between central and peripheral emetic pathways (Horn et al., 2014). Nausea and vomiting are related but distinct processes. Nausea is a subjective and unpleasant sensation that may occur without motor expulsion, whereas vomiting represents a coordinated reflex involving gastrointestinal, respiratory, abdominal, and autonomic responses (Zhong et al., 2021; Miller, 1990). This distinction is clinically relevant because nausea is often more difficult to quantify and may persist even in the absence of active vomiting. The central regulation of emesis is primarily organized through an integrated neural network within the brainstem. The nucleus tractus solitarius is considered a major integration center that receives input from several afferent pathways, including the gastrointestinal tract, vestibular system, chemoreceptor trigger zone, and higher cortical or limbic structures. These inputs are then coordinated into the autonomic and somatic responses that produce retching and vomiting (Pasricha et al., 2022; Babic and Browning, 2014; Yates et al., 2014). Peripheral gastrointestinal signaling plays an important role in PONV. Surgical manipulation, intestinal distension, mucosal irritation, inflammation, and blood or secretions within the gastrointestinal tract may stimulate vagal afferent fibers (Babic and Browning, 2014; Yates et al., 2014). Enterochromaffin cells in the gut can release serotonin, which activates 5-hydroxytryptamine type 3 receptors on vagal afferents and transmits emetogenic signals to the brainstem. This gut-brain communication is particularly relevant after abdominal, laparoscopic, and gastrointestinal procedures, although similar mechanisms may contribute to PONV across different surgical settings (Alcaino et al., 2025; Gan, 2005). Furthermore, the chemoreceptor trigger zone, located in the area postrema, is another important component of the emetic pathway. Because this region has an incomplete blood-brain barrier, it can detect emetogenic substances circulating in the blood and cerebrospinal fluid (Han and de Araujo, 2021). Anesthetic agents, opioids, metabolic disturbances, and other perioperative exposures may activate this region and contribute to nausea and vomiting through dopaminergic, serotonergic, opioid, and neurokinin-mediated signaling (Smith and Laufer, 2014; Golembiewski and O'Brien, 2002). In addition, vestibular input also contributes to emesis, particularly in patients susceptible to motion-related nausea. Signals from the vestibular apparatus are transmitted through histaminergic and muscarinic cholinergic pathways to brainstem centers involved in emetic control. Although vestibular mechanisms are not the dominant cause of PONV in all patients, they provide an important explanation for the contribution of motion, early mobilization, and individual susceptibility to postoperative nausea (Yates et al., 2014; Apfel et al., 1999; Leung and Hon, 2019). Higher cortical and limbic pathways may further modulate the perception of nausea. Anxiety, pain, unpleasant sensory stimuli, and previous negative perioperative experiences may influence nausea through interactions among cortical, limbic, hypothalamic, autonomic, and brainstem emetic networks (Varangot-Reille et al., 2023; Napadow et al., 2013; Laufenberg-Feldmann et al., 2019). These pathways help explain why nausea is not purely a gastrointestinal reflex but rather a multidimensional symptom influenced by physiological, pharmacological, and psychological factors. Several neurotransmitter systems participate in PONV, including serotonin, dopamine, histamine, acetylcholine, substance P acting on neurokinin-1 receptors, and opioid receptor-mediated pathways (Zhao et al., 2025; Weibel et al., 2020; Stegen et al., 2024; Jotaki et al., 2024) (Figure 1). The involvement of multiple receptor systems explains why PONV may persist despite single-agent therapy and provides the biological rationale for combining antiemetic agents with different mechanisms of action in patients requiring prophylaxis or treatment.

### Risk factors and prediction models

**FIGURE 2:**
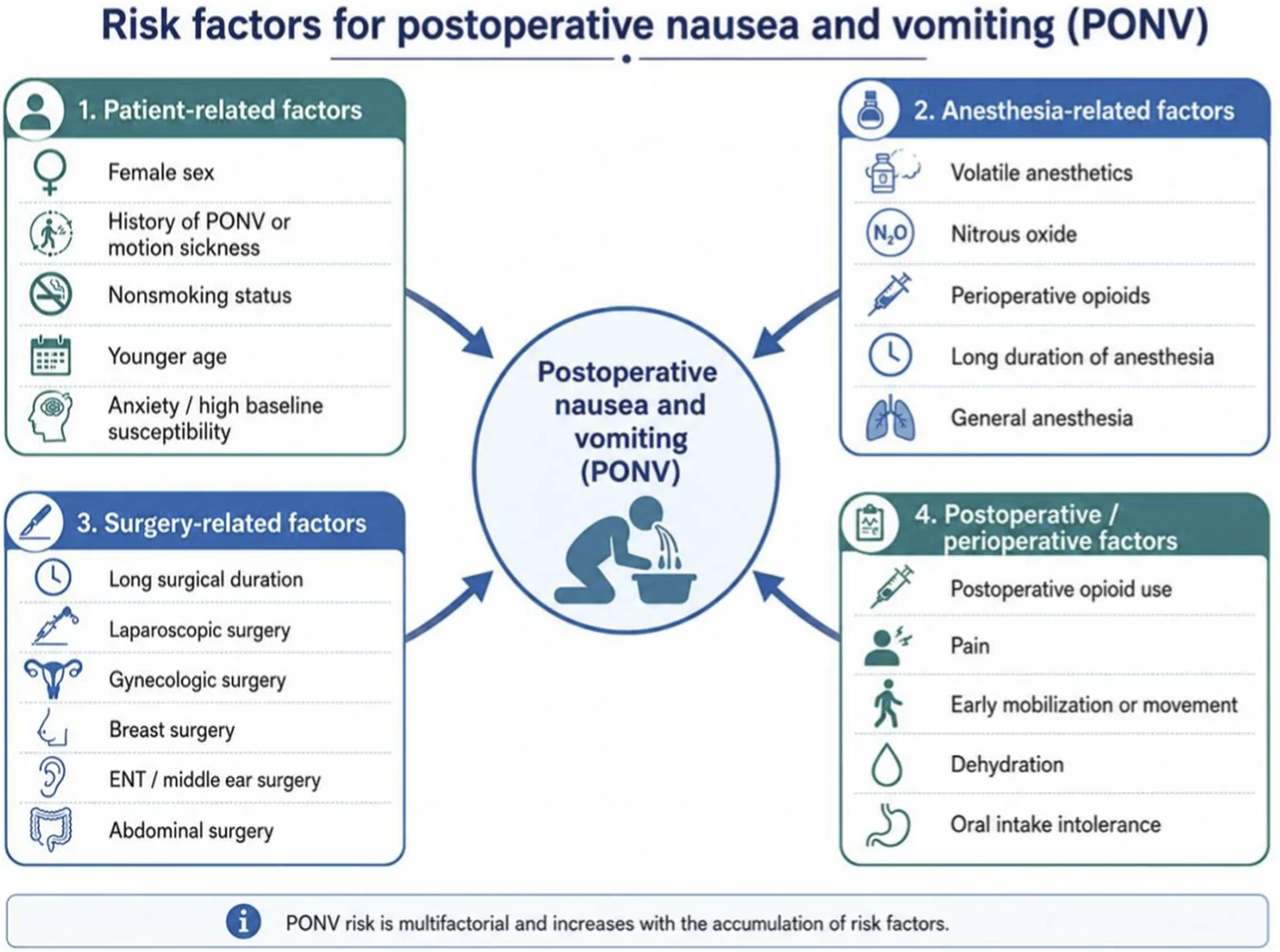
Overview of patient, anesthesia, surgery, and postoperative/perioperative risk factors contributing to postoperative nausea and vomiting.

The risk of PONV is influenced by a combination of patient-related, anesthesia-related, and surgery-related factors (Figure 2). Because no single factor can reliably predict PONV in all patients, structured risk assessment is recommended to guide preventive strategies. Risk stratification allows clinicians to identify patients who may benefit from baseline risk reduction and multimodal antiemetic prophylaxis, while avoiding unnecessary medication use in low-risk individuals (Dou et al., 2026; Murugappan et al., 2021). Patient-related factors are among the strongest predictors of PONV. In adults, female sex, nonsmoking status, previous history of PONV or motion sickness, and younger age have consistently been associated with increased PONV risk. A personal history of PONV or motion sickness is particularly important because it reflects individual susceptibility to emetogenic stimuli (Boogaerts et al., 2000; Yumuşak Ergin et al., 2026). Nonsmoking status should be regarded as an epidemiologically derived risk-prediction covariate rather than a modifiable therapeutic factor. Although smoking has been associated with a lower observed incidence of PONV, the underlying mechanism remains uncertain, and this association should not be interpreted as evidence that smoking is protective or as a basis for any clinical recommendation regarding smoking behavior (Frelich et al., 2025; Chimbira and Sweeney, 2000). Furthermore, anesthesia-related factors also contribute substantially to PONV risk (Weibel et al., 2020). Volatile anesthetic agents, nitrous oxide, longer duration of anesthesia, and perioperative opioid administration are well-recognized contributors. Opioids are particularly relevant because they may increase nausea through central emetic pathways, delayed gastric emptying, and altered gastrointestinal motility. Therefore, the need for postoperative opioid analgesia is commonly included in risk prediction models and is also considered a modifiable perioperative factor (Feng et al., 2024; Jewer et al., 2019; Peyton and Wu, 2014). In addition, surgery-related factors may further modify the likelihood of PONV. Higher rates have been reported after several procedures, including laparoscopic, gynecological, abdominal, breast, otolaryngological, and ophthalmological surgeries (Huang et al., 2023; Zhang Q. et al., 2025; Fortier et al., 2010; Joo et al., 2016; Şişman et al., 2023). However, the independent contribution of surgical type is variable and may be influenced by patient selection, anesthetic technique, operative duration, postoperative pain intensity, and opioid requirement (Son and Yoon, 2018). Therefore, surgical category should be interpreted as part of a broader risk profile rather than as an isolated determinant.

The Apfel simplified score is one of the most widely used adult prediction models for PONV. It includes four predictors: female sex, nonsmoking status, history of PONV or motion sickness, and expected postoperative opioid use (Darvall et al., 2021). In the original validation, the estimated incidence of PONV was approximately 10%, 21%, 39%, 61%, and 79% in patients with zero, one, two, three, and four risk factors, respectively. Thus, the score provides a practical quantitative framework for stratifying patients from relatively low to high PONV risk and guiding the intensity of preventive strategies (Darvall et al., 2021; Ziemann-Gimmel et al., 2020; Avinash and Krishna, 2023). Other scoring systems have also been proposed, including models that incorporate additional patient, anesthetic, or surgical variables. However, increasing model complexity does not always improve practical bedside decision-making (Zou et al., 2026; Shim et al., 2022). For this reason, simplified tools remain valuable in daily practice, especially when used as part of a structured perioperative pathway. In pediatric patients, separate prediction models are required because risk profiles differ from those in adults, with factors such as age, surgical procedure, history of PONV in the child or relatives, and postoperative opioid use being more relevant (Urits et al., 2020; Portnoy et al., 2025). Although prediction models are useful, they should not be viewed as absolute determinants of outcome. PONV remains a multifactorial complication, and individual clinical judgment is necessary when applying risk scores. The value of risk assessment lies in supporting a rational preventive plan: low-risk patients may require limited or no prophylaxis, whereas moderate- and high-risk patients usually benefit from a more comprehensive approach that combines reduction of modifiable risk factors with antiemetic prophylaxis (Jin et al., 2020). For postdischarge risk assessment, postdischarge nausea and vomiting (PDNV) is particularly relevant following ambulatory surgery. A simplified PDNV risk score identified five independent predictors: female sex, age <50 years, history of nausea and/or vomiting after anesthesia, opioid administration in the post-anesthesia care unit (PACU), and nausea in the PACU. The corresponding incidence of PDNV increased from 7% in patients with no risk factors to 20%, 28%, 53%, 60%, and 89% in those with one through five factors, respectively (Apfel et al., 2012b). This score may facilitate identification of patients who could benefit from prolonged antiemetic prophylaxis and postdischarge monitoring.

### Baseline risk reduction strategies

Baseline risk reduction represents an important step in the prevention of PONV, particularly because several perioperative contributors are potentially modifiable. While patient-related susceptibility cannot always be changed, anesthetic technique, analgesic strategy, fluid management, and perioperative care pathways can be adjusted to reduce emetogenic exposure (Pang and Chan, 2024; Hu et al., 2025). This approach is especially valuable because it addresses the underlying perioperative triggers of PONV rather than relying exclusively on pharmacological prophylaxis. One of the most effective strategies is to minimize exposure to emetogenic anesthetic agents when clinically appropriate. Volatile anesthetics and nitrous oxide are recognized contributors to PONV, especially during the early postoperative period (Li et al., 2021; Fernández-Guisasola et al., 2010). Therefore, the use of propofol-based total intravenous anesthesia may be considered in patients with elevated risk or in procedures where rapid recovery and discharge are important priorities (Keck et al., 2026; Lim et al., 2018). However, the choice of anesthetic technique should always be individualized according to surgical requirements, patient comorbidities, airway considerations, hemodynamic stability, and institutional practice (Hu et al., 2025; Dom et al., 2026).

Opioid-sparing analgesia is another central component of baseline risk reduction. Perioperative opioids can contribute to nausea and vomiting through central emetic stimulation and delayed gastrointestinal motility (Ghai et al., 2022; Zhang Z. et al., 2025). Multimodal analgesic regimens, including non-opioid analgesics, regional anesthesia, local infiltration techniques, and nerve blocks when appropriate, may reduce opioid requirements while maintaining adequate pain control (Majerić Kogler et al., 2023; Joshi, 2023). This strategy is particularly important because poorly controlled pain itself may impair recovery and increase postoperative stress; therefore, the goal is not opioid avoidance at all costs, but rather balanced analgesia with the lowest effective opioid exposure. Furthermore, regional and neuraxial anesthesia may also reduce PONV in selected patients by decreasing the need for general anesthetic exposure and systemic opioids. When suitable for the surgical procedure and patient condition, these techniques may support early recovery, improve analgesia, and reduce reliance on rescue opioid administration (Dom et al., 2026; Zhu Y. J. et al., 2025; Gan et al., 2020). Nevertheless, regional approaches should be integrated into a broader perioperative plan, as their effect on PONV may vary according to the type of block, surgical site, sedation strategy, and postoperative analgesic regimen (Dom et al., 2026; Zhu Y. J. et al., 2025; Gan et al., 2020; Moka, 2025). Adequate perioperative hydration is another supportive measure that may help reduce nausea, particularly in patients at risk of dehydration, hypotension, or delayed oral intake (Pang and Chan, 2024). Although fluid administration alone is not sufficient to prevent PONV in all patients, avoidance of hypovolemia may improve hemodynamic stability, tissue perfusion, and postoperative tolerance of oral intake (Pang and Chan, 2024; Yavuz et al., 2014; Maharaj et al., 2005). Fluid therapy should be individualized to avoid both under-resuscitation and unnecessary fluid overload (Yavuz et al., 2014). Additional perioperative measures may further support PONV prevention. These include minimizing prolonged fasting when consistent with fasting guidelines, avoiding unnecessary gastric insufflation, reducing swallowed blood or secretions when relevant, maintaining hemodynamic stability, and encouraging gradual postoperative mobilization and oral intake (Şişman et al., 2022). In enhanced recovery pathways, these interventions are typically combined with standardized analgesic, anesthetic, and antiemetic protocols to improve overall recovery quality (Jin et al., 2020; Sidik et al., 2024). Overall, baseline risk reduction should be viewed as the foundation of PONV prevention. By decreasing modifiable emetogenic exposures and optimizing perioperative care, clinicians may reduce the intensity of prophylaxis required and improve the effectiveness of antiemetic strategies.

### Pharmacological prevention and treatment

Pharmacological prophylaxis is a central component of PONV prevention, particularly in patients with moderate or high predicted risk. Because PONV is mediated by multiple neurochemical pathways, no single antiemetic agent is universally effective in all patients. Therefore, preventive therapy and treatment are commonly selected according to individual risk, expected emetogenic exposure, patient comorbidities, medication safety profile, and the need to target different receptor systems (Golembiewski and Tokumaru, 2006; Parrish et al., 2022). Several classes of antiemetic agents are used for PONV prophylaxis and treatment. These drugs act on different receptor systems involved in emetic signaling, including serotonin, neurokinin-1, dopamine, muscarinic cholinergic, histaminergic, and inflammatory pathways (Weibel et al., 2020; Gan et al., 2025; Tan et al., 2020) (Table 1). Because each class targets a distinct component of the emetic network, combination therapy is often preferred in patients with moderate or high risk of PONV. The rationale for multimodal antiemetic prophylaxis was further established by the landmark IMPACT factorial trial, which enrolled 5,199 patients at increased risk of PONV. Ondansetron, dexamethasone, and droperidol each reduced the relative risk of PONV by approximately 26%, and their effects were largely independent of one another and of baseline patient risk (Apfel et al., 2005). These findings established the principle that combining interventions with different mechanisms can provide cumulative preventive benefit, particularly in patients at increased risk of PONV.

**TABLE 1:** Pharmacological agents commonly used for prophylaxis and treatment of postoperative nausea and vomiting (Williams et al., 2021; Rajan and Joshi, 2021; Gan et al., 2022).

Class	Drug	Primary receptor target(s)	Suggested dose	Adverse effects
Serotonin antagonists	Granisetron	5-HT3-RA	0.35–3 mg IV	Constipation, headache,and dizziness
	Ondansetron	5-HT3-RA	4 mg IV	Constipation, headache, and QT prolongation
	Dolasetron	5-HT3-RA	12.5 mg IV	Constipation, headache, and QT prolongation
	Palonosetron	5-HT3-RA	0.075 mg IV	Constipation, and headache
Dopamine-antagonists	Droperidol	D3-RA, D2	0.625–1.25 mg IV	Restlessness, and QT-prolongation, sedation, extrapyramidal effects, dysphoria, and hypotension
	Amisulpride	D3-RA, D2	5 mg IV	Elevated serum prolactin
	Metoclopramide	5-HT3-RA, D2	10–20 mg IV	Extrapyramidal effects, QT-prolongation, and restlessness
	Prochlorperazine	H1-RA, D2, D3	2.5–5 mg IV (treatment)	Blurred vision, extrapyramidal effects, sedation, hypotension and dizziness
	Perphenazine	H1, α1-RA, D2, D3	8 mg/day PO	Extrapyramidal effects, sedation, and hypotension
	Haloperidol	D3-RA, D2	0.5–1 mg IV	QT-prolongation, hypotension, dysphoria, sedation, restlessness, and extrapyramidal effects
NK1 (substance P) antagonists	Aprepitant	NK1-RA	40 mg PO	Heartburn, tiredness, hypotension, hiccups, constipation, and pruritus
	Fosaprepitant	NK1-RA	150 mg IV	Heartburn, tiredness, hypotension, hiccups, constipation, and pruritus
Corticosteroids	Dexamethasone	GC receptor agonist	4–8 mg IV	Elevation of blood glucose, perineal discomfort, and exacerbation of anxiety
Anticholinergics	Scopolamine	M-RA	Transdermal patch	Dry mouth, dizziness, dry eyes, sedation, skin rash, restlessness, and pruritus
Antihistamines	Promethazine	M-RA, H1-RA	6.25–12.5 mg IV	Confusion (in elderly), severe tissue injury with extravasation, extrapyramidal reactions, dry mouth, sedation, and dry mouth
	Dimenhydrinate	M-RA (especially M5), H1-receptor inverse agonist	50–100 mg PO	Mydriasis, sedation, tachycardia, dry mouth, blurred vision, urinary retention, irritability and constipation

Abbreviations: 5-HT3, 5-hydroxytryptamine type 3 receptor (serotonin receptor); RA, receptor antagonist; D, dopamine; α, alpha-adrenergic; GC, glucocorticoid; H, histamine; M, muscarinic; NK, neurokinin; IV, intravenous; PO, oral.

### 5-HT3 receptor antagonists

The serotonin receptor system comprises seven receptor families (5-HT1–5-HT7), encompassing at least 14 receptor subtypes, among which the 5-hydroxytryptamine type 3 (5-HT3) receptor has a particularly important role in emetic signaling (Johnston et al., 2014; Zhang Y. et al., 2020). Unlike most serotonin receptors, 5-HT3 receptors are ligand-gated ion channels. Their activation produces rapid neuronal depolarization through excitatory postsynaptic potentials and promotes calcium ion influx into serotonergic neurons (Zhang Y. et al., 2020; Ye et al., 2026). This increase in intracellular calcium can facilitate the release of several emetogenic neurotransmitters and neuropeptides, including dopamine, substance P, cholecystokinin, glutamate, acetylcholine, and serotonin itself, thereby amplifying signaling within peripheral and central vomiting pathways (Faerber et al., 2007; Zhong et al., 2017). On this mechanistic basis, 5-HT3 receptor antagonists have become a major pharmacological class for both prophylaxis and treatment of PONV (Loewen et al., 2000). Ondansetron is the most extensively studied agent in this group and remains widely used in clinical practice because of its established efficacy, availability, and clinician familiarity (Tricco et al., 2015; Khoori et al., 2024). Other first-generation agents include granisetron, dolasetron, tropisetron, and ramosetron. Although these drugs share a common receptor target, they differ in pharmacokinetic properties, duration of action, receptor affinity, and clinical performance (Kovac, 2016; Gugale and Bhalerao, 2016; Roberts et al., 2012; Kim et al., 2011). Palonosetron, a second-generation 5-HT3 receptor antagonist, has several pharmacological characteristics that distinguish it from earlier agents. It has a prolonged elimination half-life of approximately 40 h and exhibits distinctive interactions with the 5-HT3 receptor, including allosteric binding, positive cooperativity, and receptor internalization. Palonosetron may also modulate crosstalk between 5-HT3 and NK1 receptor signaling pathways, without directly antagonizing NK1 receptors (Rojas et al., 2008; Rojas et al., 2014). These properties may explain its sustained antiemetic activity and its reported efficacy against both early and delayed postoperative vomiting. In addition, Comparative studies have suggested that palonosetron may provide superior protection against PONV compared with dexamethasone and several older 5-HT3 receptor antagonists (Xiong et al., 2015; Singh et al., 2016a). In previous analyses, palonosetron reduced 24-h postoperative vomiting compared with ramosetron, improved early vomiting control compared with granisetron, and was associated with lower early and 24-h vomiting rates compared with ondansetron, together with higher complete response rates (Rojas et al., 2014; Li et al., 2015a). These findings indicate that palonosetron may be particularly useful when prolonged antiemetic coverage is desired.

The adverse-effect profile of 5-HT3 receptor antagonists is generally acceptable, but safety considerations remain important. Reported adverse reactions include headache, dizziness, constipation, tachycardia, QTc prolongation, and, rarely, serotonin syndrome (Tricco et al., 2016; Goodin and Cunningham, 2002). Palonosetron appears to have a lower likelihood of clinically meaningful QTc prolongation compared with some older agents (Apfel and Jukar-Rao, 2012). By contrast, ondansetron carries a warning from the United States Food and Drug Administration regarding QTc prolongation and the potential risk of serious cardiac events (Singh et al., 2023). Therefore, while ondansetron remains a practical and widely used antiemetic, palonosetron may offer advantages in selected patients because of its longer duration of action, favorable efficacy profile, and potentially lower cardiac conduction risk.

## Dopamine receptor antagonists

Dopaminergic signaling is an important component of the emetic pathway. Experimental evidence has shown that dopamine D2 and D3 receptors participate in the regulation of nausea and vomiting, particularly within central structures involved in emetic control (Belkacemi and Darmani, 2020). On this basis, dopamine receptor antagonists have been used clinically for both prophylaxis and treatment of PONV, especially when symptoms are triggered by multiple emetogenic stimuli or when prophylaxis with other antiemetic classes is insufficient (Wolfe and Bequette, 2021). Clinically used dopamine D2-like receptor antagonists include several pharmacological groups. Butyrophenones, such as haloperidol, have established antiemetic activity and may be used at low doses for PONV prevention or rescue therapy (Lam et al., 2024). Phenothiazines, such as perphenazine, also exert antiemetic effects through dopamine receptor blockade, although their use may be limited by sedation and other central nervous system effects (Homburger, 1958; Williams et al., 2021). Benzamides, including amisulpride and metoclopramide, represent another clinically relevant group, with amisulpride being increasingly recognized as an option for established PONV, including cases that occur despite prophylaxis (Zhong et al., 2021; Zhang L. F. et al., 2020). Commonly used doses include haloperidol 0.5–1 mg, with an approximate duration of action of 3–4 h; amisulpride 5 mg; and droperidol 0.625–1.25 mg (Rajan and Joshi, 2021; Gan et al., 2022). Drug selection should consider local availability, institutional protocols, patient risk factors, and whether the agent is being used for prophylaxis or rescue treatment. Although dopamine receptor antagonists are effective antiemetics, their adverse-effect profile requires careful attention. Excessive dosing may cause pronounced sedation, hypotension, extrapyramidal reactions, acute dystonia, akathisia, or other movement-related adverse effects (Jenkins, 2024; Orhan et al., 2024). Recent observational studies have also explored the use of oral perphenazine as a preoperative antidopaminergic agent. Because it has relatively limited sedative effects at antiemetic doses, an established safety record in large clinical observations, and no remaining patent-related restriction, perphenazine may represent a practical option in selected multimodal prophylactic regimens (Belkacemi and Darmani, 2020; Williams et al., 2021; Henao et al., 2014). Nevertheless, additional high-quality comparative studies are needed to define its optimal role in contemporary PONV prevention.

### NK1 receptor antagonists

Neurokinin-1 (NK1) receptors are distributed in both central and peripheral structures involved in the emetic reflex, including brainstem regions and gastrointestinal-associated neural pathways (Saito et al., 2003; Darmani and Ray, 2009). Substance P, the endogenous ligand of the NK1 receptor, is a major mediator of vomiting and contributes to emetic signaling through activation of this receptor system (Darmani and Ray, 2009; Andrews et al., 2025). NK1 receptor antagonists exert their antiemetic effect by competitively blocking substance P binding, thereby interrupting one of the key pathways involved in nausea and vomiting (Andrews et al., 2025). Aprepitant is the most widely recognized NK1 receptor antagonist and was initially established for the prevention and treatment of nausea and vomiting associated with highly emetogenic chemotherapy (Ibrahim and Preuss, 2026; Zhu H. et al., 2025). Its clinical application has since expanded to PONV, where evidence supports its efficacy, particularly in reducing vomiting and delayed emetic symptoms (Weibel et al., 2020). Because NK1 receptor signaling differs from serotonergic, dopaminergic, histaminergic, and cholinergic pathways, aprepitant may be especially useful as part of multimodal prophylaxis in patients at increased risk of PONV. Clinical studies have demonstrated that aprepitant provides antiemetic benefit in surgical patients (Grigio et al., 2025). In a large controlled trial involving 922 patients undergoing abdominal surgery, oral aprepitant at doses of 40 or 125 mg showed non-inferior complete response rates compared with ondansetron during the first 24 h after surgery. More importantly, aprepitant was superior to ondansetron in preventing vomiting during both the 0–24 h and 0–48 h postoperative periods, and it also showed favorable effects on nausea control (Diemunsch et al., 2007). These findings were subsequently reinforced by a systematic review and meta-analysis of 12 randomized trials involving 2,729 patients, which showed that aprepitant significantly reduced the incidence of PONV compared with ondansetron alone (12.5% vs. 28.5%; RR, 0.45; 95% CI, 0.29–0.72) and improved complete response rates (RR, 1.13; 95% CI, 1.03–1.24), with trial sequential analysis indicating conclusive evidence for the reduction in overall PONV incidence (Singh et al., 2024). Current consensus recommendations support the use of oral aprepitant 40 mg before induction of anesthesia for PONV prevention in appropriate patients (Gan et al., 2020). Its long duration of action makes it particularly attractive for high-risk patients, ambulatory surgery, and situations in which postoperative vomiting could cause important clinical consequences (Gan et al., 2020). However, routine use may be influenced by cost, availability, oral administration requirements, and potential drug interactions. Therefore, NK1 receptor antagonists should be selected according to patient risk, surgical context, and the overall antiemetic strategy.

### Glucocorticoids

Glucocorticoids are known agents in the prevention of PONV and are frequently incorporated into multimodal antiemetic regimens. Their precise antiemetic mechanism has not been fully clarified; however, several mechanisms have been proposed, including suppression of perioperative inflammatory mediator release, modulation of prostaglandin activity, reduction of serotonin release, and possible direct effects on central emetic pathways (Lavand’homme and Kehlet, 2023; Yang et al., 2017). Dexamethasone is the most commonly used glucocorticoid for PONV prophylaxis because of its efficacy, low cost, long duration of action, and familiarity in anesthetic practice. Current guideline-based dosing commonly recommends intravenous dexamethasone at 4–8 mg, usually administered near induction of anesthesia to allow adequate onset of action (Gan et al., 2020). Methylprednisolone has also been used as an alternative, with a commonly recommended intravenous dose of 40 mg (Gan et al., 2020). Beyond its antiemetic effect, perioperative dexamethasone may provide additional recovery-related benefits. Intraoperative administration has been associated with improved control of acute postoperative pain and may contribute to reduced analgesic requirements in selected patients (Corcoran et al., 2023; Impiumi and Kearsley, 2025). This analgesic-sparing effect may indirectly support PONV prevention by reducing the need for postoperative opioids. Therefore, glucocorticoids remain valuable antiemetic agents, but their use should be individualized according to patient comorbidities, infection risk, glycemic status, and the overall perioperative plan (Corcoran et al., 2021; Chu et al., 2014).

### Anticholinergics

Cholinergic signaling is involved in several emetic pathways, particularly those related to vestibular input and central autonomic regulation (Zhang and Burger, 2024). Acetylcholine may contribute to nausea and vomiting through muscarinic receptor activation, and drugs that increase cholinergic activity may also provoke emetic symptoms in susceptible patients (Li et al., 2015b; Eisenman, 2009). Anticholinergic agents reduce this signaling by antagonizing muscarinic receptors, thereby limiting cholinergic transmission within pathways involved in nausea and vomiting. Scopolamine is the most widely used anticholinergic agent for nausea and vomiting prevention, especially in motion sickness and vestibular-mediated nausea (Apfel et al., 2010; Spinks and Wasiak, 2011). Its transdermal formulation provides prolonged drug delivery and has demonstrated efficacy in the prevention of PONV (Apfel et al., 2010; Pergolizzi et al., 2012). In addition, penehyclidine, another anticholinergic agent, has also been reported to reduce PONV in clinical settings (Zhao et al., 2024). These agents may be particularly useful when prolonged protection is needed or when vestibular mechanisms are expected to contribute to postoperative symptoms. Although scopolamine can be effective as a single adjunctive agent, some evidence suggests that it may reduce the benefit of certain combined antiemetic strategies, including regimens involving aprepitant or dopamine receptor antagonists (Weibel et al., 2020). Therefore, anticholinergics should not be added automatically to all prophylactic protocols; instead, their use should be individualized according to patient risk factors, expected duration of symptoms, contraindications, and the overall antiemetic plan. Despite their antiemetic benefit, anticholinergic drugs require careful patient selection because of their adverse-effect profile. Common side effects include dry mouth, blurred vision, dizziness, constipation, urinary retention, and sinus tachycardia (Collamati et al., 2016).

### Antihistamines

Histamine exerts its biological effects through four receptor subtypes, among which the H1 receptor is most closely associated with vestibular-mediated nausea and vomiting (Chen et al., 2018; Parsons and Ganellin, 2006). Activation of H1 receptors within brainstem emetic pathways is considered an important mechanism in motion sickness-related vomiting (Chen et al., 2018). Accordingly, H1 receptor antagonists, including promethazine and diphenhydramine, have long been used for the treatment of nausea and vomiting associated with vestibular stimulation and motion sickness (Zhong et al., 2021; Schaefer et al., 2026).

In the context of PONV, however, antihistamines are not commonly used as first-line monotherapy (Jin et al., 2020). Their prophylactic and therapeutic roles are generally more limited than those of 5-HT3 receptor antagonists, glucocorticoids, dopamine receptor antagonists, and NK1 receptor antagonists (Jin et al., 2020; Gan et al., 2025). This limitation is partly related to their sedative properties, which may delay postoperative recovery, impair early mobilization, and reduce discharge readiness, particularly in ambulatory surgery. Recent observational studies have suggested that low-dose intravenous diphenhydramine, commonly within the range of 12.5–20 mg, may have value as part of multimodal antiemetic prophylaxis (Williams et al., 2021; Williams et al., 2011; Williams et al., 2023; Lin et al., 2005). At these doses, diphenhydramine may provide antihistaminic benefit while avoiding excessive sedation in selected patients. It has also been proposed that low-dose diphenhydramine may partially replace other sedating agents used in perioperative regimens, such as dexmedetomidine, although its antiemetic potency may be less pronounced (Williams et al., 2021; Williams et al., 2011; Williams et al., 2023). The potential benefit of diphenhydramine in multimodal PONV prevention may not be explained solely by H1 receptor blockade. Because diphenhydramine also has mild anticholinergic activity, its antiemetic effect may reflect combined antihistaminic and muscarinic modulation. This dual action may be clinically useful when administered before surgery, provided that the dose remains low enough to minimize anticholinergic adverse effects such as dry mouth, urinary retention, confusion, or delayed gastrointestinal recovery (Williams et al., 2023).

### Additional pharmacological approaches

Additional pharmacological agents have also been investigated for PONV prevention. Olanzapine has demonstrated prophylactic antiemetic efficacy, with a recent systematic review and meta-analysis showing a reduction in PONV compared with control (RR, 0.62; 95% CI, 0.42–0.90), although the relatively small evidence base warrants cautious interpretation (Grigio et al., 2024). Gabapentinoids, including gabapentin and pregabalin, have been associated with a modest reduction in postoperative nausea and vomiting; however, their routine perioperative use remains controversial because of limited overall clinical benefit and an increased risk of adverse effects such as dizziness and visual disturbance (Verret et al., 2020). Perioperative benzodiazepines, particularly midazolam, may also exert antiemetic effects; a recent systematic review and meta-analysis demonstrated a significant reduction in PONV (RR, 0.77; 95% CI, 0.66–0.89), supporting their potential role as part of multimodal prophylaxis in selected patients (Au et al., 2024). Dexmedetomidine, an α2-adrenergic receptor agonist, has similarly been associated with a reduced incidence of PONV in adults undergoing general anesthesia, potentially through anesthetic- and opioid-sparing effects, although adverse effects such as bradycardia should be considered (Zhao et al., 2023).

## Rescue treatment of established PONV

Established postoperative nausea and vomiting requires prompt treatment because persistent symptoms may delay oral intake, mobilization, and discharge and may adversely affect postoperative recovery. Selection of rescue therapy should be guided by whether prophylactic antiemetics were administered, the pharmacological classes previously used, the interval since administration, and the expected duration of action of those agents. Importantly, evidence for rescue treatment is substantially less extensive than that for prophylaxis. A systematic review specifically examining rescue therapy identified 45 randomized controlled trials and highlighted considerable uncertainty regarding the optimal drug, dose, route, and timing of treatment. Nevertheless, current consensus recommendations provide a practical framework for managing both antiemetic-naïve patients and patients in whom prophylaxis has failed (Gan et al., 2025; Gan et al., 2022). The central principle for breakthrough PONV after failed prophylaxis is to administer an antiemetic with a different mechanism of action from the prophylactic agent or agents previously given. Re-administration of an antiemetic from the same pharmacological class generally provides little additional benefit during the early postoperative period. This principle is supported by randomized evidence showing that repeating intravenous ondansetron after failed ondansetron prophylaxis was not more effective than placebo, as well as by subsequent evidence syntheses. Accordingly, if a patient who received a 5-HT3 receptor antagonist develops PONV, rescue treatment should preferentially involve another class, such as a dopamine receptor antagonist or an antihistaminic/anticholinergic agent when clinically appropriate. If more than 6 h have elapsed since prophylaxis and no suitable alternative is available, current consensus guidance allows consideration of a second dose of a short-acting 5-HT3 receptor antagonist or a butyrophenone (Gan et al., 2025; Gan et al., 2022; Kovac et al., 1999). Clinical evidence also supports several specific rescue options. In patients who had failed ondansetron prophylaxis, promethazine was more effective than repeat ondansetron treatment; a large retrospective analysis reported complete response rates of 68% with promethazine compared with 50% following repeat ondansetron. Promethazine 6.25 mg appeared as effective as higher doses, whereas increasing the dose was associated with greater concern regarding sedation, supporting use of the lowest effective dose (Gan et al., 2022; Habib et al., 2007). Intravenous amisulpride represents another evidence-based option, particularly when previous prophylaxis did not include a dopamine antagonist. A multicenter randomized phase III trial demonstrated that intravenous amisulpride 10 mg was effective for established PONV after failure of prophylaxis, and current consensus recommendations recognize 10 mg as the more appropriate rescue dose in patients who previously received a non-antidopaminergic prophylactic regimen (Gan et al., 2025; Habib et al., 2019). Amisulpride at 5 or 10 mg has also demonstrated efficacy for treatment of established PONV in patients who received no prior prophylaxis (Candiotti et al., 2019).

For antihistamine-naïve patients who did not receive prophylaxis, treatment may be selected from established antiemetic classes according to patient characteristics and contraindications. Evidence synthesized in the recent consensus guideline indicates that droperidol 1–1.25 mg has rescue efficacy comparable to ondansetron 4–8 mg, whereas metoclopramide 10 mg appears less effective than either ondansetron or droperidol. Low-dose propofol (20–40 mg) has also demonstrated antiemetic activity, although its short duration of action and sedative effects limit its role to closely monitored settings such as the post-anesthesia care unit (Gan et al., 2025; Gan et al., 2022). These comparative findings should be interpreted cautiously because the certainty of rescue-specific evidence for several agents remains low to moderate. Re-dosing decisions should additionally account for drug duration of action. Long-acting agents such as aprepitant, fosaprepitant, and palonosetron should generally not be repeated in the immediate recovery period. Dexamethasone is likewise not routinely re-administered for breakthrough PONV because of its prolonged pharmacological effect, while transdermal scopolamine is unsuitable for immediate rescue because its onset of action is approximately 2–4 h. Thus, an appropriate rescue algorithm consists of identifying the prophylactic agents already administered, selecting a rapid-onset antiemetic from a different pharmacological class, avoiding unnecessary same-class re-dosing during the first 6 h, and considering the pharmacokinetic profile and adverse-effect burden of each candidate agent. This class- and time-based approach provides a rational framework for individualized treatment of established PONV (Gan et al., 2025; Gan et al., 2022).

## Current limitations

Despite substantial progress in the understanding and management of PONV, several important limitations remain. First, although current risk prediction models are simple and clinically useful, their predictive accuracy is imperfect. Most available tools rely on a limited number of clinical variables and may not fully capture the complexity of individual susceptibility, anesthetic exposure, surgical context, psychological factors, and postoperative care pathways. As a result, some patients classified as low or moderate risk may still develop clinically significant PONV, whereas others may receive prophylaxis without clear benefit (Jin et al., 2020; Apfel et al., 2001; Wu et al., 2015). Second, the evidence base for PONV prevention and treatment remains heterogeneous. Studies differ in surgical population, anesthetic technique, antiemetic regimen, timing of assessment, outcome definitions, and follow-up duration (Weibel et al., 2020; Gabby et al., 2021). Some investigations focus primarily on vomiting, whereas nausea severity, patient distress, postdischarge symptoms, and quality of recovery are less consistently evaluated (Singh et al., 2016b; Myles and Wengritzky, 2012). This variability makes direct comparison between studies difficult and may limit the generalizability of findings across different clinical settings. Another important limitation is the incomplete translation of guideline-based recommendations into routine practice. Although risk assessment, baseline risk reduction, and multimodal prophylaxis are widely recommended, implementation may be inconsistent because of time constraints, lack of standardized institutional protocols, medication availability, cost considerations, and variability in clinician preference.

## Future directions

**FIGURE 3:**
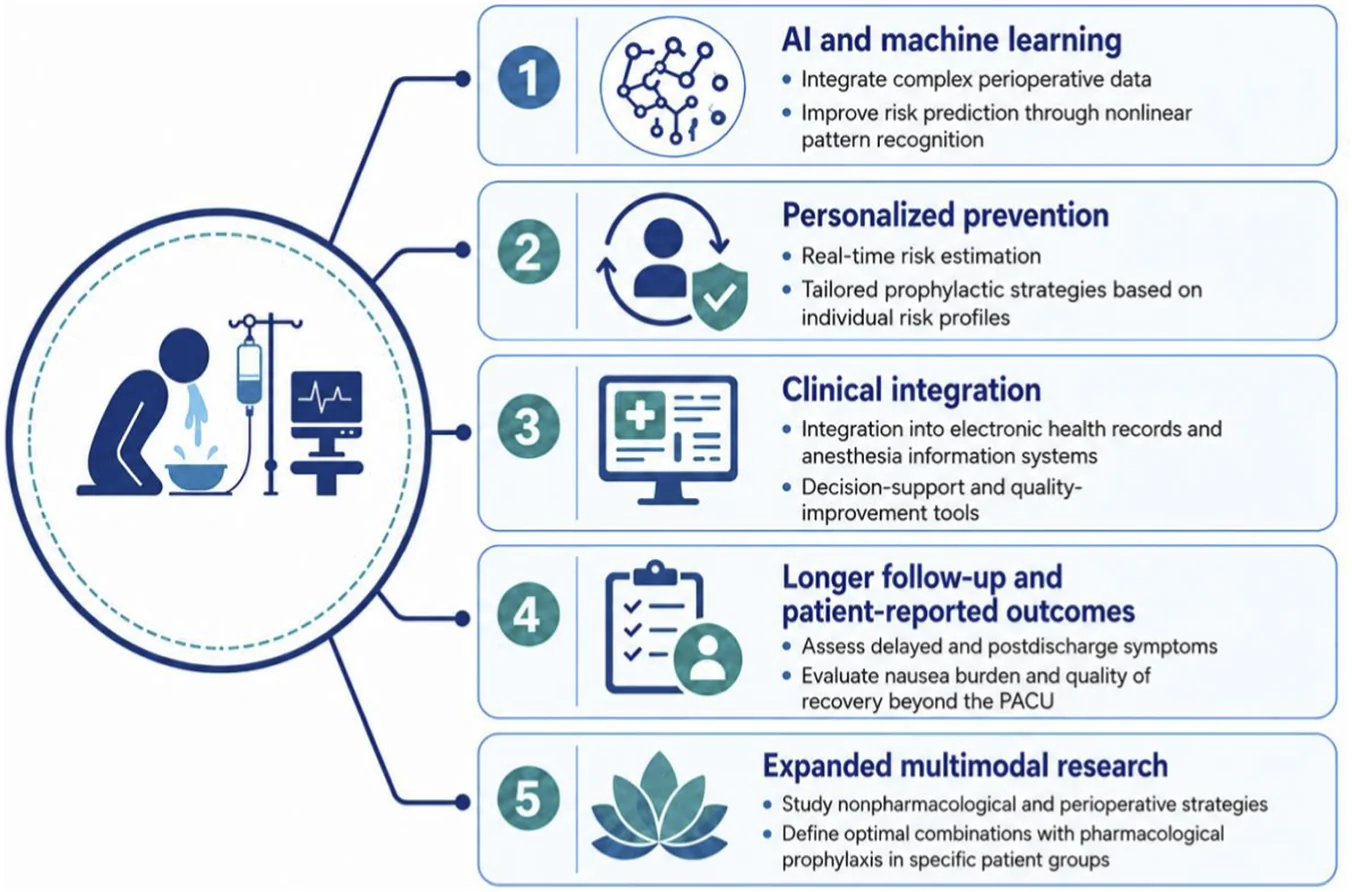
General overview of key future directions for improving PONV prediction, prevention, clinical implementation, follow-up assessment, and multimodal management.

Future efforts should focus not only on identifying effective antiemetic strategies but also on improving adherence through practical perioperative pathways, electronic decision-support tools, and quality-improvement programs (Kooij et al., 2008; Kooij et al., 2010; Choy et al., 2022) (Figure 3). Artificial intelligence and machine learning represent promising future directions for improving PONV prediction and individualized prevention. Compared with traditional risk scores, AI-based models may incorporate larger and more complex datasets, including demographic characteristics, comorbidities, anesthesia records, intraoperative physiological variables, medication exposure, surgical information, and postoperative recovery data (Shim et al., 2022; Xie et al., 2023; Zheng et al., 2024). These models may help identify nonlinear relationships and interactions that are difficult to detect using conventional scoring systems (Shim et al., 2022; Xie et al., 2023). In the future, AI-assisted prediction tools could be integrated into electronic health records or anesthesia information systems to provide real-time risk estimation and guide personalized prophylactic strategies (Xie et al., 2023; Fan et al., 2025). However, the clinical use of AI in PONV management remains at an early stage. Many available models are developed from retrospective or single-center datasets, and their performance may decline when applied to different hospitals, surgical populations, anesthetic protocols, or healthcare systems.

Furthermore, postdischarge nausea and vomiting remains an under-recognized problem, particularly in ambulatory surgery. Many studies assess symptoms only during the early recovery period, although nausea and vomiting may occur after discharge and negatively affect hydration, oral medication intake, sleep, mobility, and patient satisfaction (Carroll et al., 1995; Wu et al., 2002; Gupta et al., 2003). Future research should include longer follow-up intervals and patient-reported outcomes to better evaluate delayed symptoms and the real-world burden of PONV beyond the post-anesthesia care unit. In addition, nonpharmacological and perioperative care strategies also deserve further investigation. Approaches such as acupuncture-related techniques, optimized fasting and hydration protocols, opioid-sparing analgesia, regional anesthesia, and enhanced recovery pathways may reduce PONV when integrated into comprehensive perioperative care (Jewer et al., 2019; Lee et al., 2025; Cheong et al., 2013; Nam et al., 2024). Future research should determine how these strategies interact with pharmacological prophylaxis and whether specific combinations provide additional benefit in defined patient groups (Figure 3). Furthermore, a prospective observational study identified significant differences in preoperative gut microbiota between patients with and without PONV, including reduced microbial diversity and lower Bifidobacterium abundance in affected patients, suggesting a potential role of the gut microbiome in PONV susceptibility. Further studies are needed to determine whether microbiome-related mechanisms, including gut-brain signaling and interactions with perioperative drug metabolism, could improve PONV risk prediction and individualized prevention (Tang et al., 2025).

## Conclusion

PONV remains an important perioperative complication that requires early risk recognition, reduction of modifiable triggers, and individualized multimodal prevention. Effective management should integrate anesthetic planning, opioid-sparing analgesia, appropriate antiemetic selection, and timely rescue treatment. Future work should focus on optimizing prophylactic combinations, improving postdischarge symptom control, and strengthening the practical implementation of evidence-based perioperative pathways.
